# Distribution of *Aedes aegypti* and *Aedes albopictus*, and the current situation of dengue fever and chikungunya in Iran and neighboring countries: a review study

**DOI:** 10.1371/journal.pntd.0013965

**Published:** 2026-02-11

**Authors:** Mohammad Shoushtari, Hasan Bakhshi, Mostafa Salehi-Vaziri, Morteza Zaim, Ahmadali Enayati, Mohammad Hassan Pouriayevali, Ghobad Moradi, Mohammad Mahdi Sedaghat, Abdolreza Mirolyaei, Ehsan Mostafavi, Fahimeh Bagheri Amiri

**Affiliations:** 1 Department of Virology, Pasteur Institute of Iran, Tehran, Iran; 2 Department of Arboviruses and Viral Hemorrhagic Fevers (National Reference Laboratory), Research Centre for Emerging and Reemerging Infectious Diseases, Pasteur Institute of Iran, Tehran, Iran; 3 Department of Medical Entomology and Vector Control, School of Public Health, Tehran University of Medical Sciences, Tehran, Iran; 4 Department of Medical Entomology and Vector Control, School of Public Health and Health Sciences Research Center, Mazandaran University of Medical Sciences, Sari, Iran; 5 Department of Epidemiology and Biostatistics, Faculty of Medicine, Kurdistan University of Medical Sciences, Sanandaj, Iran; 6 Center for Communicable Diseases Control, Ministry of Health and Medical Education, Tehran, Iran; 7 Department of Epidemiology and Biostatistics, WHO Collaborating Center for Vector-Borne Diseases, Research Centre for Emerging and Reemerging Infectious Diseases, Pasteur Institute of Iran, Tehran, Iran; Mizan-Tepi University, ETHIOPIA

## Abstract

**Introduction:**

*Aedes*-borne diseases, such as dengue and chikungunya, are public health threats worldwide. Due to climate change and the expansion of *Aedes* mosquitoes, several countries are reporting the local transmission of *Aedes*-borne arboviruses. In 2024, Iran faced a significant rise in the number of imported dengue cases and the first local transmission of the disease in the southern provinces of Hormozgan and Sistan and Baluchistan. This review summarizes the latest data on the distribution of invasive *Aedes* mosquitoes and the epidemiological status of dengue fever and chikungunya in Iran and neighboring countries.

**Methods:**

A comprehensive search was carried out on papers and reports concerning epidemiological records and studies on dengue fever, chikungunya, *Aedes aegypti (Ae. aegypti)*, and *Aedes albopictus* (*Ae. Albopictus)*, as well as the recent situation in Iran and neighboring countries since 2000. Meanwhile, the epidemiological trend and milestones of these arboviruses and their vectors in Iran and their last updates in neighboring countries were assessed.

**Results:**

In addition to Iran, at least nine neighboring countries including Armenia, Turkey, Iraq, Afghanistan, Pakistan, the United Arab Emirates, Oman, Qatar, and Saudi Arabia have reported the establishment of *Ae. aegypti* and/or *Ae. albopictus* mosquitoes. Local dengue virus transmission was reported in Iran, Pakistan, Afghanistan, Oman, the United Arab Emirates, and Saudi Arabia. However, the local circulation of chikungunya virus was only reported in Pakistan.

**Conclusion:**

The establishment of *Ae. aegypti* in southern Iran (Hormozgan, Sistan and Baluchistan, Bushehr) and *Ae. albopictus* in northern/northwestern provinces (Guilan, Mazandaran, Ardabil, East Azerbaijan, Zanjan, Qazvin) has created distinct arbovirus transmission risks. Local dengue outbreaks in 2024 were exclusively reported in *Ae. aegypti*—infested areas (Chabahar, Bandar Lengeh), correlating with this vector's known efficiency in urban transmission. While chikungunya remains undocumented in local mosquito populations, serological evidence and recent report of the infected non-*Aedes* species suggest potential cryptic circulation. With climate models predicting habitat expansion for both vectors, Iran's emerging *Aedes*-borne diseases’ burden could escalate if no action is planned. This underscores the imperative for integrated surveillance targeting mosquito distributions, human case trends, and cross-border pathogen flow to mitigate outbreak risks.

## 1. Introduction

Arboviruses, or arthropod-borne viruses, are a group of viruses transmitted to humans and animals through blood-feeding arthropods such as mosquitoes and ticks [1]. The most notable arboviruses affecting humans belong to three families: *Togaviridae*, *Flaviviridae*, and *Peribunyaviridae* [2,3]. Among these, dengue virus (DENV) and chikungunya virus (CHIKV), belonging to the *Flaviviridae* and *Togaviridae* families, respectively, are recognized as the most prevalent and impactful arboviral infections worldwide [4,5]. Recently, the incidence of diseases caused by DENV and CHIKV has increased dramatically, particularly in tropical and subtropical regions, including the Middle East, raising serious public health concerns [6,7]. The primary transmission route for DENV and CHIKV involves infected female mosquitoes of the Culicidae family, particularly *Aedes aegypti* and *Aedes albopictus*, which serve as efficient vectors for both arboviruses in tropical and subtropical regions [8].

These viruses can cause debilitating symptoms and, in severe cases, may lead to mortality, underscoring the urgent need for effective control strategies. The complex transmission dynamics of DENV and CHIKV pose significant challenges for health authorities, necessitating targeted interventions to mitigate outbreaks.

DENV and CHIKV have serious public health impacts with similar epidemiological behavior and clinical symptoms [9]. As of September 2024, global data reports indicate over 13 million dengue cases, with more than 8,500 dengue-related deaths [10]. Additionally, there are approximately 460,000 cases of chikungunya, resulting in 170 CHIKV-related deaths [11]. Data from 1979 to 2022 show that around 2.5 billion people worldwide live in climate-suitable zones for DENV transmission [12]. Projections suggest that, due to climate change and urbanization, the number of at-risk individuals could rise to 6.1 billion by 2050 [13].

The DENV has four serotypes (DENV-1, -2, -3, and -4), each capable of causing infection and inducing type-specific (homotypic) lifelong immunity. While many DENV infections are asymptomatic or result in only mild illness, the infection can also lead to severe forms of dengue, and even death, mainly due to secondary infections with a different serotype [14]. While CHIKV infection is generally not fatal, it can cause chronic arthralgia/arthritis and leads to long-term complications that threaten patients’ overall health [6,15]. In addition to chronicity, severe infections, CHIKV and DENV pose increased risks of hospitalization and mortality in vulnerable populations. These viruses also affect the economy through absenteeism and associated social and economic losses [16,17]. On the other hand, DENV and CHIKV overload the healthcare systems during outbreaks and epidemics [18]. The first documented report of dengue fever in Iran was an imported case identified in 2008, with recent travel history to Kuala Lumpur, Malaysia [19]. Subsequently, local transmission was confirmed in 2024 following an outbreak that included 865 autochthonous cases (852 in Chabahar and 12 in Bandar Lengeh) associated with imported cases. In this outbreak, a large number of dengue cases were identified with a history of traveling to the United Arab Emirates (UAE) [20].

This review provides an overview of the recent evidence on the geographic distribution of *Ae. aegypti* and *Ae. albopictus* and summarizes the current epidemiological situation of dengue fever and chikungunya in Iran and its neighboring countries.

## 2. Methods

Covering an area of 1,648,195 km^2^, Iran is the 18th-largest country in the world and the second largest in the Middle East [21]. Iran borders Armenia, Azerbaijan, and Turkmenistan to the north. Kazakhstan and Russia are other neighbors of Iran by the Caspian Sea. Iran is bordered by Turkey and Iraq to the northwest and west, and by Afghanistan and Pakistan to the east. The southern boundary, stretching 1,770 km (1,100 miles), is formed by the coastlines of the Persian Gulf and the Gulf of Oman. Bahrain, Kuwait, Oman, Qatar, the UAE, and Saudi Arabia are maritime neighbors of Iran to the south.

In this review, we searched PubMed and Google Scholar databases to collect the data published during 2000–2024, using logical operators to combine the relevant keywords including: “dengue”, OR “chikungunya”, OR “*Aedes*”, OR “*Aedes aegypti*”, OR “*Aedes albopictus*”, the name of each countries including: “Iran”, “Iraq”, “Turkey”, “Azerbaijan”, “Armenia”, “Turkmenistan”, “Afghanistan”, “Pakistan”, “Kuwait”, “Saudi Arabia”, “Bahrain”, “Qatar”, “United Arab Emirates”, and “Oman”, to collect the desired articles. Although the strategy primarily focused on published documents, when no evidence was found in scientific articles, Google website (www.google.com) was also used to purposefully search for official government reports and verified news sources using a backward-step time approach. Additionally, we incorporated reports from the World Health Organization (WHO), the Iranian Center for Disease Control and Prevention (ICDC), and other relevant national health authorities to enrich our data on disease prevalence and vector distribution in Iran and neighboring countries.

The inclusion criteria were: (1) studies conducted in Iran or its neighboring countries, and (2) studies focusing on *Ae. aegypti* and/or *Ae. albopictus*, as well as on DENV and/or CHIKV. In line with the primary objective of the study, we reviewed all epidemiological research concerning humans and invasive *Aedes* mosquitoes in Iran, along with the latest conditions in neighboring countries.

## 3. Results

### 3.1. Data collection

Our systematic literature search across PubMed and Google Scholar databases initially retrieved 2,398 articles relevant to Iran and its neighboring countries. Based on the study objectives, records related to Iran were screened systematically, whereas records from other countries were screened purposively using a backward-step time approach.

After screening for regional relevance and removing duplicates, the dataset was narrowed down to 112 records specifically focused on Iran. Among them, full-text evaluation of 77 articles led to the final inclusion of 14 studies that met our criteria: (1) epidemiological data on *Ae. aegypti* and/or *Ae. albopictus* distribution, and (2) evidence of DENV and/or CHIKV transmission patterns in Iran. For neighboring countries with limited published research, official government reports, and verified news sources were also included.

### 3.2. Distribution of *Ae. aegypti* and *Ae. albopictus* mosquitoes in Iran and neighboring countries

#### 3.2.1. Iran.

**Fig 1:**
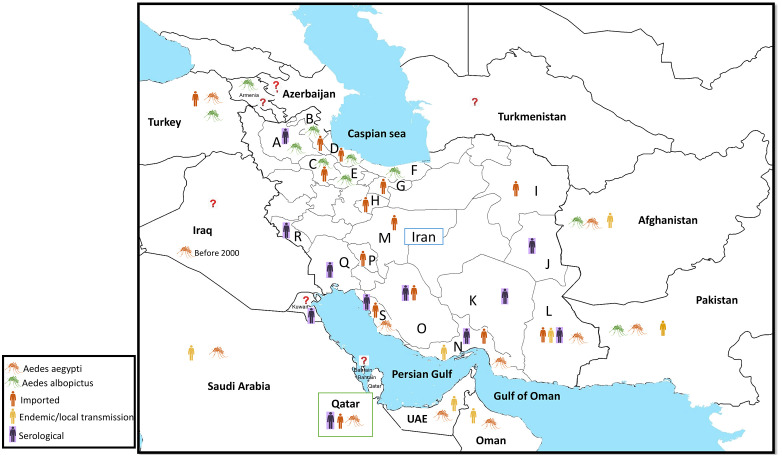
Geographic distribution of dengue fever and the distribution of *Aedes aegypti* and *Aedes albopictus* mosquitoes in Iran and its neighboring countries. The letters correspond to the provinces of Iran as follows: A: East Azerbaijan, B: Ardabil, C: Zanjan, D: Guilan, E: Qazvin, F: Mazandaran, G: Tehran, H: Qom, I: Khorasan Razavi, J: South Khorasan, K: Kerman, L: Sistan and Baluchistan, M: Isfahan, N: Hormozgan, O: Fars, P: Chaharmahal and Bakhtiari, Q: Khuzestan, R: Ilam, S: Bushehr. The **?** shows that we couldn’t find evidence about the occurrence of *Ae. aegypti* and *Ae. albopictus* mosquitoes in Turkmenistan, Azerbaijan, Kuwait, and Bahrain; nor the reports of DENV in Iraq, Armenia, Turkmenistan, Azerbaijan, and Bahrain. Geographical map of Iran generated using QGIS (version 3.34; http://www.QGIS.org). Administrative boundary shapefiles for Iran were obtained from the National Cartographic Center (https://en.ncc.gov.ir/), and global shapefiles were sourced from Esri’s World Countries (Generalized) dataset (https://hub.arcgis.com/datasets/esri::world-countries-generalized/explore).

**Fig 2:**
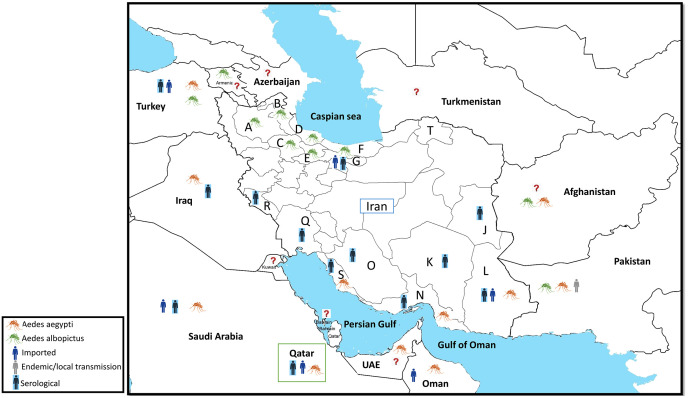
Geographic distribution of chikungunya and the distribution of *Aedes aegypti* and *Aedes albopictus* mosquitoes in Iran and neighboring countries. The geographic distribution of 57 imported cases, reported until December 10, 2024, is not shown on this map. Alphabets show the name of Iran provinces: A: East Azerbaijan, B: Ardabil, C: Zanjan, D: Guilan, E: Qazvin, F: Mazandaran, G: Tehran, J: South Khorasan, K: Kerman, L: Sistan and Baluchistan, N: Hormozgan, O: Fars, Q: Khuzestan, R: Ilam S: Bushehr and T: North Khorasan. The **?** shows that we couldn’t find evidence about the occurrence of *Ae. aegypti* and *Ae. albopictus* mosquitoes in Turkmenistan, Azerbaijan, Kuwait, and Bahrain; nor the reports of CHIKV in Armenia, Turkmenistan, Azerbaijan, Kuwait, Bahrain, and UAE. Geographical map of Iran generated using QGIS (version 3.34; http://www.QGIS.org). Administrative boundary shapefiles for Iran were obtained from the National Cartographic Center (https://en.ncc.gov.ir/), and global shapefiles were sourced from Esri’s World Countries (Generalized) dataset (https://hub.arcgis.com/datasets/esri::world-countries-generalized/explore).

Entomological investigations on *Aedes* mosquitoes in Iran began after the first report of an imported dengue fever case in 2008. According to the latest data from the ICDC, *Ae. aegypti* has been collected in three provinces located in southeastern and southern Iran: Hormozgan, Sistan and Baluchistan, and Bushehr [22]. The record of *Ae. albopictus* in the northern part of Iran was reported in Guilan province in 2023 [23] (**Table 1**). Additionally, based on ICDC report, *Ae. albopictus* mosquitoes have been collected in six provinces located in northern and northwestern parts of Iran: Guilan, and a limited area of Mazandaran, Ardabil, East Azerbaijan, Zanjan, and Qazvin [22] (**Figs 1** and **2**).

**Table 1:** Entomological studies that provide evidence of the occurrence of *Aedes albopictus* and *Aedes aegypti* in Iran from 2008 to 2024.

First author	Province(s)	Year(s) of finding	No. of the collected samples	Type of the trap	Type of habitat of trapped *Aedes* sp.	The invasive species identified	Reference
Doosti S.	Sistan and Baluchistan, Hormozgan, Bushehr, Fars, Kerman, Khuzestan, Ilam, South Khorasan	2008–2014	Five larvae; seven adults	Dipper, dropper, human bait	Small and shallow ditch, plastic bottle, and earthenware, near the solid dam	*Ae. albopictus*	[24]
Yaghoobi-Ershadi, M. R.	Sistan and Baluchistan	2012–2014	Seven adults	Aspirator, pyrethrum spray space catch	Bed net and shade of trees	*Ae. albopictus*	[25]
Azizi K. Dorzaban H	Hormozgan	2016–2020	47 larvae; eight adults	Dipping, aspirator, net trap	Different habitats	*Ae. aegypti*	[26,27]
Azari-Hamidian Sh	Guilan	2023	29 larvae that were reared from collected eggs; 896 adults	Aspirator, light trap, BG lure trap, ovitrap	Not defined	*Ae. albopictus*	[23]

#### 3.2.2. Neighboring countries.

*Ae. aegypti* and *Ae. albopictus* have been reported in at least nine neighboring countries of Iran. In the eastern border of Iran, *Ae. albopictus* and *Ae. aegypti* have been reported in Pakistan (2024) [28] and Afghanistan (2019) [29]. No information was available for the northern neighboring countries of Iran, except for Armenia (2021) [30,31]. Furthermore, *Ae. aegypti* has been reported in both Western bordered countries, Iraq (based on the reports before 2000) [32], and Turkey [33]. Turkey has also reported the presence of *Ae. albopictus* [34]. Among the countries bordering the Persian Gulf, no documented publications were found from the UAE, despite reports from various websites indicating the presence of *Ae. aegypti* in 2024 [35]. Notably, epidemiological surveillance data confirmed that a substantial proportion of imported dengue cases in Iran in 2024 were linked to travel history to the UAE [20], suggesting the possible presence of unreported *Aedes* populations in the country. Saudi Arabia (2023) [36], Oman (2018–2019) [37,38], and Qatar (1999) [39] have reported the presence of *Ae. aegypti*. However, to our knowledge, no information is available for Bahrain. In addition, although some publications claim that “there is no evidence of the existence of *Aedes* mosquitoes in Kuwait [40], travel advisories for Kuwait still mention a risk of DENV transmission [41] (**Fig 1**).

### 3.3. Distribution of DENV

#### 3.3.1. DENV in Iran.

The first imported dengue case in Iran was laboratory-confirmed in July 2008 by the National Reference Laboratory for Arboviruses at Pasteur Institute of Iran, Tehran, in a 61-year-old male patient with recent travel history to Malaysia [19]. After that, imported cases in Iran were reported annually [42]. Serological studies conducted on symptomatic individuals in Iran between 2012 and 2017 have reported the presence of dengue fever in Fars, Khuzestan, Ilam, Hormozgan, Bushehr, Kerman, Sistan and Baluchistan, and South Khorasan provinces [43–46]. In a study conducted in Hormozgan Province in 2016–2017, none of the collected *Ae. aegypti* mosquitoes were positive for DENV [26]. In addition, serological studies during 2020–2023 on blood donors of Hormozgan, Bushehr, Khuzestan, Sistan and Baluchistan, Kerman, Guilan, East Azarbaijan, and West-Azarbaijan provinces showed a history of infection [47,48] (**Table 2**). Between 2017 and 2024, an average of 20 imported dengue cases were reported each year; however, there was a notable surge in reported cases, with a total of 1076 positive cases recorded from May 15, 2024, to Jan 12, 2025. Among the recorded cases, 211 cases reported a history of travel to several countries including UAE, Pakistan, Oman, and Benin [22]. There were 865 patients without a history of international travel, 13 of them were infected in Bandar Lengeh (Hormozgan Province) and 852 in Chabahar (Sistan and Baluchistan Province) indicating local transmission. Twelve cases of patients with a history of traveling to Chabahar (local transmission) have been identified and diagnosed in Ardabil, Chaharmahal and Bakhtiari, Sistan and Baluchistan (Zahedan), and Isfahan [22] (Fig 1 and Table 2). None of these investigations confirmed seropositive samples using additional neutralization assays such as PRNT, leaving the possibility of cross-reactivity with other flaviviruses unaddressed [49].

**Table 2:** Epidemiological studies on symptomatic and asymptomatic DENV and CHIKV Iranian population during 2000–2024.

First author	Studied population(s)	Province(s)/Region(s)	Year of study	Type of detection	Positive cases/total sample	Positive cases (%) with travel history/no travel history)	Reference
**DENV**							
Chinikar S.	Patients negative for CCHF	All regions of the country	2000–2012	IgG and IgM/confirmed by PCR	3/300 confirmed	All had a history of travel	[43]
					12/ 300 just serology	Five positive cases had a history of travel.	
Heydari	Clinically suspected patients	Sistan and Baluchistan	2013–2015	IgG/IgM	7/60	None of the patients had abroad travel history	[44]
				NS1	2/60		
Tavakoli F.	Patients with fever and rash, but with negative measles and rubella IgM.	Fars, Khuzestan, Ilam, Kerman, Hormozgan, Bushehr, Kerman, Sistan and Baluchistan, South Khorasan	2016–2017	IgM	82/1306	Not assessed	[45]
Khalili M.	Febrile patients	Kerman, Sistan and Baluchistan, South Khorasan	2016	IgG/NS1 and PCR	0/184	Not assessed	[46]
Aghaie A.	Blood donors	Sistan and Baluchistan	–	IgG/IFA	32/540	None of the positive people had traveled abroad	[47]
Seyed-Khorami	General population	Hormozgan, Bushehr, Khuzestan, Sistan and Baluchistan, Kerman, Guilan, East Azarbaijan, West-Azarbaijan	2020–2023	IgG	256/11192	16.4 Vs. 2.3%	[48]
**CHIKV**							
Tavakoli F	Patients with fever and rash, but with negative measles and rubella IgM	Fars, Khuzestan, Ilam, Hormozgan, Bushehr, Sistan and Baluchistan, Kerman, South Khorasan	2016–2017	IgM	210/1306	Not assessed	[45]
Seyed-Khorami SM.	General population	Hormozgan, Bushehr, Khuzestan, Sistan and Baluchistan, Kerman Province from south of Iran, and Guilan, East Azarbaijan and West-Azarbaijan	2020–2023	IgG	11/11192	0% Vs. 0.1%	[48]
Pouryavali MH	patients suspected of CHIKV	Sistan and Baluchistan	2017–2018	Serology or Molecular	40/159	65.6%TVs. 0.0%	[62]
Solgi A.	Outpatient kids	Tehran	2018	IgG	4/180	Not assessed	[63]
Podin M.	Symptomatic patients	Sistan and Baluchistan	2020	IgG	3/42	Not assessed	[64]
	Not symptomatic				0/161		

#### 3.3.2. DENV in neighboring countries.

The local transmission of DENV has been documented in Pakistan (2024) [50], Afghanistan (2019) [29], Oman (2022–2023) [51,52], and Saudi Arabia (2023) [53]. Except for one report of imported cases in 2019 [54], there is no formal documented evidence of the disease in the UAE. However, informal and indirect sources (2023 and 2024) suggest local circulation of DENV in the country [35,55]. Additionally, 150 out of 221 imported cases in Iran, reported up to October 2024, had a history of travel to the UAE [20]. In a study conducted in southern Iraq (2012–2013), DENV was not detected [56]. A case of suspected dengue fever in Armenia in 2023 was reported without laboratory confirmation [57]. Several imported cases have been reported in Turkey, with the most recent case in 2019 [58]. Serological evidence of dengue fever has been reported in Qatar (2013–2019) [39,59] and Kuwait (2022) [60]. To our knowledge, no evidence of circulation of DENV has been found in Bahrain, Azerbaijan, and Turkmenistan (**Fig 1**).

### 3.4. Distribution of CHIKV

#### 3.4.1. CHIKV in Iran.

According to the ICDC, from 2016 to Jan 2025, nearly 60 imported cases of CHIKV have been reported in Iran [22,61]. Serological evidence from studies on symptomatic patients (2017–2020) has confirmed CHIKV infection in several provinces, including Fars, Khuzestan, Ilam, Hormozgan, Bushehr, Sistan and Baluchistan, Kerman, South Khorasan, and Tehran [45,62–64] (Table 2). Moreover, a serological study conducted between 2020 and 2023 provided evidence of CHIKV exposure among the general population of Sistan and Baluchistan Province, indicating that infections have also occurred in community-representative residents without symptom-based selection [48]. In a study conducted in 2018, a total of 1,212 mosquitoes were analyzed, and CHIKV of the Asian genotype was detected in six pools (*Culiseta longiareolata*, *Culex tritaeniorhynchus*, and *Anopheles maculipennis* s.l.), using a high-throughput screening method. These infected non-*Aedes* mosquito species were collected from North Khorasan and Mazandaran provinces, located in the north of Iran (**Fig 2 and Table 2**). This marks the first report of these mosquitoes infected with CHIKV [65]. Among the infected species, the *Cs. longiareolata* pools contained engorged mosquitoes. This species may have become infected after blood feeding on viremic individuals returning from Pakistan [66].

#### 3.4.2. CHIKV in neighboring countries.

Among the studied countries, evidence of local circulation of CHIKV has been documented in Pakistan [67]. However, to our knowledge, there are no reports of this arbovirus in Bahrain, Afghanistan, Azerbaijan, Armenia, and Turkmenistan. Although there is no direct report of CHIKV in the UAE, a study conducted in Thailand in 2018–2019 identified some imported cases linked to travel to the UAE. Based on this, the investigators classified the UAE as a country with possible ongoing transmission [68]. The published data on the situation of CHIKV in Saudi Arabia suggest that this arbovirus is not endemic in that country [69]. Serological evidence and/or imported cases of chikungunya have been reported in Iraq (2012–2013) [56], Turkey (2015) [70,71], Oman (1999–2013) [72,73], and Qatar (2013–2016) [39,59]. While no published document was found on CHIKV in Kuwait during 2000–2024, using references list of a systematic review [74], we found an old and out-of-search period serological evidence of infection to CHIKV in Kuwait about five decades ago (1979–1982) [75].

## 4. Discussion and conclusion

In this study, we thoroughly reviewed the occurrence of two invasive *Aedes* mosquitoes, as well as two important *Aedes*-borne viral diseases in Iran and its neighboring countries. Our results indicate that *Ae. aegypti* and *Ae. albopictus* have been reported in nine countries, in addition to Iran. According to the literature and the ICDC reports until January 2025, *Ae. albopictus* and *Ae. aegypti* have been reported in various provinces of Iran. *Ae. albopictus* has primarily been detected in northern and northwestern provinces, including Guilan [23], Mazandaran, Ardabil, East Azerbaijan, Zanjan, and Qazvin [22]. In contrast, *Ae. aegypti* has been reported in southern provinces such as Hormozgan [26,27], Sistan and Baluchistan, and Bushehr [22]. This distribution suggests that both vector species have adapted to the various climatic zones within the country. This is in line with modeling studies predicting the future distribution of *Aedes* mosquitoes. These modeling and scenarios suggest that suitable habitats for *Ae. albopictus* may gradually shift to the northwest of Iran, while southern regions provide suitable habitats for *Ae. aegypti.* Climate change projections indicate the potential expansion of these habitats into southwestern and northeastern regions by 2030 [76]. Concurrently, while no locally transmitted cases were reported in Iran as of July 2024, subsequent epidemiological investigations revealed an increase in imported cases from dengue-endemic neighbors (particularly the UAE), combined with expanding *Aedes* mosquitoes populations in southern coastal regions, precipitated local transmission outbreaks. The port city of Chabahar in Sistan and Baluchistan Province has been identified as the primary emergence zone [20].

In many of Iran’s neighboring countries, despite the prevalence of mosquito-borne diseases such as DENV and CHIKV, integrated and effective control policies have not been implemented. Iran and these neighboring countries, which are at risk, could enhance their ability to manage DENV and CHIKV epidemics by establishing early warning networks. CHIKV, already endemic in countries like Pakistan, shares epidemiological similarities with DENV. This, combined with the widespread distribution of *Aedes* mosquitoes, suggests that local transmission of CHIKV in Iran could follow a pattern similar to DENV.

The policy options for preventing and controlling dengue fever in the country, prioritized according to three national scenarios, focuses on optimally combining interventions, strengthening surveillance and reporting systems, enabling rapid response, and improving multi-level health infrastructure with trained personnel [77]. But optimizing prevention of dengue and chikungunya outbreaks will require complementary next-generation approaches. To contextualize Iran’s response, it is necessary to examine current national control strategies in the context of international recommendations. This situation underscores the urgent need for more effective and coordinated vector control measures, especially at airports and border crossings, to prevent further spread and reduce the risk of future outbreaks, as the economic burden of *Aedes*-borne diseases requires immediate intervention [78]. Meanwhile, Iran's national *Aedes* control program has adopted a multi-component integrated vector management (IVM) strategy. The proposed IVM strategy for Iran includes three synergistic components. First, an enhanced surveillance system needs to be implemented using WHO-standardized methodologies. This will involve systematic mosquito monitoring through ovitraps and larval surveillance at sentinel sites. Parallel human serological surveys are also needed to be conducted. Second, genomic surveillance involves routine sequencing of viral isolates. Third, a carefully phased implementation of novel biological controls could be considered in the long term, starting with pilot release of *Wolbachia*-infected mosquitoes in selected urban areas, contingent upon adequate capacity development at regional reference laboratories, before any potential broader application. This multidimensional framework provides a robust platform for proactive and adaptive disease management. Meanwhile, the Iranian Ministry of Health has initiated a set of targeted interventions to mitigate *Aedes*-borne diseases through three primary pathways: First, by enhancing intersectoral collaboration to prevent mosquito establishment in non-infested regions. Second, deploying community-level vector control programs in affected areas, combining public awareness initiatives with local environmental sanitation. Third, engaging medical universities to reinforce provincial capacities for vector surveillance and response. Routine Surveillance must be strengthened in provinces where local transmission has been reported or is suspected. Provinces like Sistan and Baluchistan, and Hormozgan are not only ecologically suitable for vector breeding due to warm, humid climates and presence of water storage practices, but are also geographically close to dengue-endemic countries such as Pakistan and Saudi Arabia. Their status as major entry points for travelers and migrant populations further elevates the risk, making them key targets for intensified vector surveillance and control.

Our review study indicates the evidence of local transmission of DENV in Iran [22], Pakistan [50], Afghanistan [29], Oman [51,52], Saudi Arabia [53], and UAE [35,55]. Meanwhile, the evidence of local circulation of CHIKV has been reported in Pakistan [67]. The most effective method for controlling these two arboviruses is the management of their *Aedes* vectors [79]. There are some limitations to the use of insecticides, resulting in insecticide resistance, environmental pollution, and related concerns, along with high long-term costs [80], so biological interventions are considered globally. Beyond vector control, an understanding of the economic burden will help justify and prioritize interventions. Although standard measures such as insecticide use and larval source reduction are widely implemented, their effectiveness is often undermined by factors such as urbanization, travel, and limited resources. Moreover, these strategies typically overlook community engagement and educational components, which are crucial for sustainability [81]. Therefore, appropriate knowledge, attitude, and practice (KAP) among all stakeholders [82], including the general population, municipalities, and healthcare workers (HCWs) to effectively manage clinical cases. In Iran, we found three KAP studies [83–85]. The results in HCWs revealed gaps in some dimensions of KAP, especially in knowledge about symptoms, prevention and control, transmission, and clinical management of dengue fever. Although attitude and practice were generally good, regular, targeted, and continuous training programs that consider regional differences and the specific needs of healthcare are needed.

Given the risks posed by the spread of dengue and chikungunya in the region, it is recommended to actively participate in international collaboration programs focusing on the surveillance, prevention, and treatment of mosquito-borne diseases. These collaborations should involve the exchange of epidemiological data, coordination of control strategies, and sharing of experiences with advanced countries. Moreover, Iran is considered an endemic country for some flaviviruses such as West Nile virus [86], which can lead to false positive results in dengue serological tests due to cross-reaction. It is suggested that all seropositivity results should be confirmed by additional tests. In conclusion, strengthening vector control measures, enhancing regional cooperation, and incorporating innovative surveillance and biological interventions are essential steps for effectively managing the risk of *Aedes*-borne diseases in Iran and neighboring countries.

## 5. Limitations of the study

This study conducted a qualitative comparison and analysis of data for Iran and its neighboring countries. It would have been beneficial to investigate the health policies of these countries to provide a more comprehensive understanding of the regional dynamics for dengue, chikungunya, and their vectors. Exploring these policies could reveal how they influence public health outcomes, particularly to concerning the management and control of dengue and chikungunya. Such an examination would enhance the study’s findings and offer valuable insights into best practices that could be adopted across borders.
